# Fiber Enrichment of 3D Printed Apricot Gel Snacks with Orange By-Products

**DOI:** 10.3390/gels9070569

**Published:** 2023-07-12

**Authors:** Carmen Molina-Montero, Diana Vicente-Jurado, Marta Igual, Javier Martínez-Monzó, Purificación García-Segovia

**Affiliations:** i-Food Group, FoodUPV, Universitat Politècnica de València, Camino de Vera s/n, 46022 Valencia, Spain; mamomon3@doctor.upv.es (C.M.-M.); diaviju@doctor.upv.es (D.V.-J.); marigra@upvnet.upv.es (M.I.); xmartine@tal.upv.es (J.M.-M.)

**Keywords:** waste, fiber, orange by-product, printing precision

## Abstract

Concern about food waste has become a major global concern. The waste generated by the agri-food industry poses an environmental challenge. However, the development of 3D printing technology offers an opportunity to address this problem. By incorporating food waste into inks, it can create personalized food tailored to individual needs. The aim of this study is the valorization of orange by-products (OB_P_) in 3D printed gels to obtain a final product in the form of a fiber-enriched snack. Gelatin gels were printed with different concentrations of apricot pulp (30, 50, and 70%) and OB_P_ was added. These gels were subjected to a freeze-drying process. The rheology of the gels before and after printing, the printing precision, and the post-treatment of the freeze-dried product, including color, shear force, and the presence of bioactive compounds, were evaluated. The addition of OB_P_ resulted in an increase in the elasticity (997–1242u) of the samples and improved the printability of them. However, an increase in the hardness (173–184u) was observed in the freeze-dried samples. The use of OB_P_ not only improves the printability of the gels but also enables obtaining fiber-enriched snacks, which could contribute to the reduction in food waste and the promotion of healthy and sustainable food.

## 1. Introduction

Food waste has become one of the major concerns worldwide, and the waste generated by the agri-food industry represents a significant challenge for the environment [1]. In pursuit of sustainable development, it is crucial to reduce food waste and valorize waste in order to ensure food and nutritional security for the entire population. Therefore, addressing the problem of food waste requires immediate action [2].

As food production increases to meet the growing demand of an ever-increasing population, greater quantities of agri-food waste will inevitably be generated. However, much of the waste generated in the agri-food industry contains compounds of high nutritional value, presenting an opportunity to reintroduce them into the value chain and promote a circular economy [3]. This valorization strategy seeks to maximize the use of resources, minimizing environmental impact and promoting sustainability.

Globally, approximately one-third of the food produced is wasted or lost. The highest wastage rates are in the range of 40–50% for tubers, fruits, and vegetables; 35% for fish; 30% for cereals; and 20% for oilseeds, meat, and dairy products [4]. In the fruit and vegetable food industry, large amounts of food waste are generated that are rich in nutrients and bioactive compounds such as vitamins, proteins, phenolics, carotenoids, and enzymes [2,5]. Orange peel is a by-product of the citrus sector with a significant content of fiber and bioactive compounds [6]. It is the main waste generated during orange juice processing, and between 8 and 20 million tons of orange waste are produced each year, with more than 50% in the form of peel [7,8].

Waste generated in the food industry can be put into other food uses such as the formulation of novel foods. Emerging 3D printing technology can play an important role in this goal by efficiently utilizing by-products and developing high-value novel foods. These wastes can be added to 3D printing food inks. However, incorporating wastes into 3D printing food inks can present challenges, as some fiber-rich wastes can negatively affect printability, i.e., on the ink’s handling and deposition capabilities during the 3D printing process, as well as its ability to maintain its structure after printing [1,9]. If this challenge can be overcome, printed foods could have prebiotic benefits by being enriched with fiber, which may positively influence the gut microbiota and have beneficial effects on metabolic, neurological, cardiovascular, and autoimmune diseases and cancer prevention [10].

On the other hand, 3D printing offers the possibility to develop customized and visually appealing foods. This enables the development of new foods with rich ingredients such as food waste fractions, which otherwise would not be considered desirable [1,11]. Several studies have investigated the use of 3D printing for waste revaluation. Muthurajan et al. [2] developed 3D printed noodles from potato and wheat flour waste. Similarly, Leo et al. [12] developed 3D printed snacks with the incorporation of orange peel, obtaining high consumer acceptance. Functional 3D printed cookies were also developed with grape pomace [13] and Feng et al. [14] developed a 3D printed yam snack with the incorporation of by-products obtained from potato processing. These advances demonstrate the potential of 3D printing to take advantage of food waste and create value-added foods.

The objective of this study was to incorporate orange by-product (OB_P_) into a 3D printed apricot pulp-based snack, with the purpose of valorizing the wastes and obtaining a final product with a fiber content of 3%. The intention was to create a snack that would function as a source of fiber, in accordance with Regulation No. 1924/2006 of the European Parliament and of the Council, issued on 20 December 2006, which regulates nutrition and health claims made on foods [15]. In addition, the printability and rheological properties of the snack were taken into account.

## 2. Results and Discussion

### 2.1. Rheological Behavior of Gels before 3D Printing

In food 3D printing, the material to be printed must have a non-Newtonian viscoelastic rheological behavior (ability to exhibit both viscous and elastic properties when subjected to load or deformation) that enables adequate viscosity to flow through the nozzle during extrusion, while at the same time having a high enough viscosity to melt with the layers during deposition [16]. Table 1 presents the viscoelastic parameters obtained from the oscillatory test performed on control gels (G30, G50, and G70) and gels with orange by-product addition (GB_P_30, GB_P_50, and GB_P_70) before 3D printing. It was observed that all samples showed similar elastic modulus (G′) and complex modulus (G*), higher than the viscous modulus (G″). In addition, the tan loss values (Tan δ) were less than 1 in all samples. Therefore, all samples showed a predominantly elastic behavior, with more solid-like properties.

**Figure 1:**
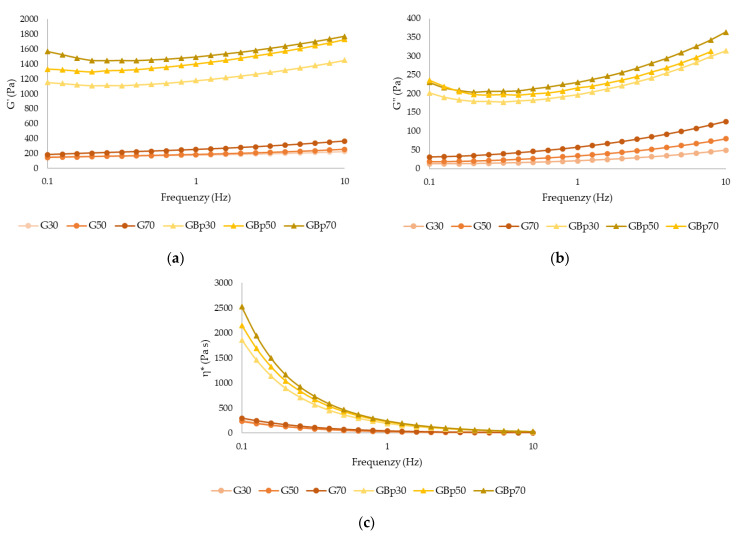
(**a**) G′, storage modulus; (**b**) G″, loss modulus; (**c**) η*, complex viscosity of the gels (with and without orange by-product) before 3D printing. Data for samples G30, G50 and G70 are extracted from Molina et al. [18] (G, gel; GBp: gel with by-product; 30, 50, and 70% apricot pulp, respectively).

Figure 1 illustrates the elastic modulus (G′), viscous modulus (G″), and apparent viscosity (η*) of control and OB_P_ samples before 3D printing. Figure 1a,b showed that an increase in pulp content in the control samples resulted in an increase in both G′ and G″ values, with no significant differences (*p* > 0.05) in G′ between samples. However, significant differences (*p* < 0.05) were found between samples when orange by-product was added, with G′ and G″ being higher than in the control samples. Furthermore, in the OB_P_ samples, an increase in the value of G′ was observed as the percentage of pulp increased and the by-product content decreased, with GB_P_70 being the sample with the highest value of G′. Similarly, in Figure 1c, an increase in viscosities was observed as the pulp content increased and orange by-product was added to the samples, and significant differences (*p* < 0.05) were found between them. All samples showed a decrease in viscosity as the frequency increased. Therefore, a change in viscoelastic behavior is observed when adding orange by-product and increasing the percentage of apricot pulp, resulting in higher fiber content. This phenomenon is attributed to the ability of fiber, especially present in apricot pulp, to absorb a significant amount of water from the sample, which causes swelling of the hemicellulose. This swelling has an effect on viscosity and gel-forming ability [14,17]. Consequently, samples containing orange by-product (3.56%) and a higher percentage of apricot pulp (70%) exhibit higher G′ values and higher viscosities.

### 2.2. Gels Behavior during 3D Printing

In food 3D printing, it is essential to understand the entire process to ensure the quality and accuracy of the final result. This is because material properties can change throughout the process, and these changes need to be taken into account [19]. The extrusion test measures the force required for the piston to extrude the mass through the printing nozzle [20]. The compression curves obtained provide crucial information on the maximum force needed to print each sample, as well as the specific requirements of the extruder [21]. The results obtained are presented in Table 2, where the maximum force (F_M_), mean force (F_m_), and gradient (Gr) values are detailed for each sample evaluated. This test plays a fundamental role in the optimization of 3D food printing parameters to ensure good printability in the production of 3D printed foods.

The gradient values obtained on the samples tested ranged from 375 N/s to 526 N/s. It was observed that the lower these values, the higher the pressure required to initiate flow, making it difficult to print the sample through the nozzle. Compared to the control samples, it was observed that the OB_P_ samples had lower gradient values, and significant differences (*p* < 0.05) were found with the control sample from a concentration of 50% apricot pulp. It was identified that the GB_P_70 sample presented the greatest difficulty to be printed. On the other hand, in the control samples, it was observed that an increase in the percentage of apricot pulp resulted in a higher F_M_, finding a clear significant difference (*p* < 0.05) in sample G70 with the rest. In the OB_P_ samples, no significant differences (*p* > 0.05) were found between them, but significant differences (*p* < 0.05) were found in the pairs G50–GB_P_50 (161–195 N) and G70–GB_P_70 (228–197 N) in terms of F_M_. It was observed that F_m_ was also influenced by increasing of pulp in the samples and by the addition of by-product, with higher values in the samples with by-product, except for sample GB_P_70.

The extrusion of the samples was influenced by both their pulp content and their orange by-product content. Higher pulp concentration and lower orange by-product concentration resulted in lower gradients and higher F_M_, which was observed in the samples with higher viscosities, GB_P_50 and GB_P_70. These samples presented greater difficulties to be printed due to gel firmness and stiffness.

### 2.3. Printability

#### 2.3.1. Behavior Rheological after 3D Printing

The rheological behavior of printing inks plays a key role both before and after the 3D printing process. Crucial information is obtained about the changes that occur during 3D printing, in addition to providing information about the structure and stability of the printed food [22,23].

**Figure 2:**
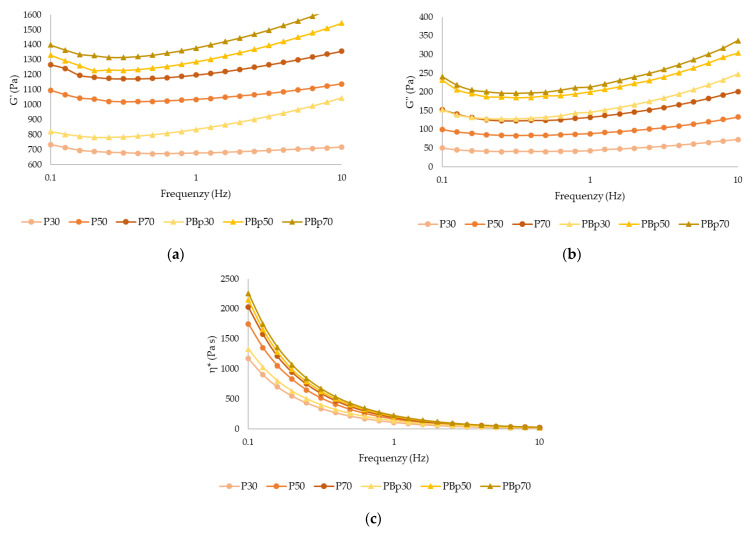
(**a**) G′, storage modulus; (**b**) G″, loss modulus; (**c**) η*, complex viscosity of the gels (with and without orange by-product) after 3D printing. (P, printed sample; PBp: printed sample with by-product; 30, 50 and 70% apricot pulp, respectively).

Table 3 shows the rheological parameters of control samples and OB_P_ samples after 3D printing. In Figure 2a,b it is observed that G′ is considerably higher than G″, and Tan δ < 1 in all samples, therefore has an elastic behavior. In the control samples, there is an increase in G′ as the pulp content in the formulations increases. Significant differences (*p* < 0.05) were found when orange by-product was added, with G′ being significantly higher than their respective control samples, except for G30–GB_P_30. Figure 2c shows the apparent viscosity presented by the different samples. As the frequency range (Hz) increased, there was a decrease in the apparent viscosity, indicating the pseudoplastic behavior of the samples, with GB_P_70 being the sample with the highest viscosity.

After printing, an increase in the rheological parameters (G*, G′, G″, and η*) was observed in the control samples, except for Tan δ, which had lower values, indicating a higher strength of the printed material. On the contrary, in the OB_P_ samples, a decrease in modulus values (G*, G′, and G″) and apparent viscosity (η*) was observed. This can be attributed to the reduced fiber content in the control sample, which facilitates homogeneous gel formation, and also the pressure exerted by the plunger increases the interconnectivity of the triple helix bond zones per molecular chain, resulting in a denser and more compact gel network [24]. In contrast, OB_P_ samples have a higher fraction of fiber, mainly insoluble. The fiber affects the gel matrix, making it difficult for the proteins to form bonds and weakens the network, resulting in a loose fragile structure [11]. Therefore, when the material is printed, the pressure exerted by the plunger breaks the three-dimensional gel network more easily, leading to a decrease in rheological parameters. These findings are consistent with a study by Alqahtani et al. [25] who reported a decrease in G′ values when oats with different particle sizes were added to gelatin. Furthermore, it was observed that sample PB_P_30, which contained a higher proportion of orange by-product and a lower apricot pulp content, had a higher fiber content, which resulted in a significant decrease of 350 units in the G′ value. On the other hand, samples PB_P_50 and PB_P_70 showed a decrease of 100 units in the G′ value, indicating better resistance to deformation.

The presence of higher fiber content in the gel decreases the stiffness of the printed material, which may have important implications for the stability and mechanical properties of 3D printed products. This can be attributed to the fact that during the printing process, it is probably the pressure applied may favor the alignment of the fibers, thus decreasing the formation of crosslinks and resulting in a softer gel.

Consequently, we can conclude that after the extrusion process of the material, the rheological properties are changed. Control gels become stiffer, while OB_P_ gels (fiber-based gels) become less stiff.

#### 2.3.2. Printing Precision of 3D Printed Gels

Dimensional stability in 3D food printing is often determined through shape fidelity. This involves measuring the extent to which the printed food structure resembles the intended design in terms of shape and form [26]. Consequently, high printability enables the fabrication of geometrically complex constructions, which increases the applicability of 3D printed foods, allowing the creation of food with customized shapes and controlled textures [27].

**Figure 3:**
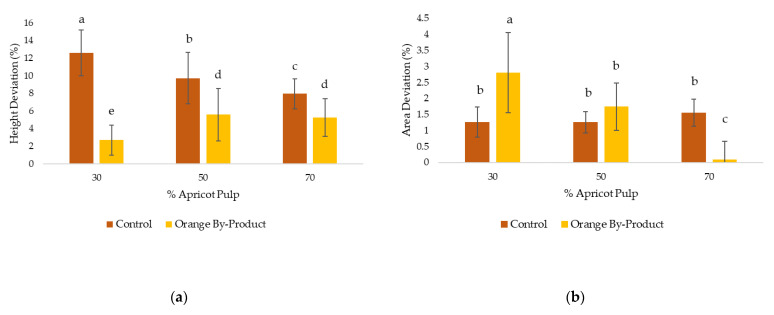
(**a**) Deviations of the height parameter of the printed samples; (**b**) deviations of the area parameter of the printed sample with respect % apricot pulp (30, 50 and 70%). Letters (a–e) indicate homogeneous groups according to ANOVA (*p* < 0.05). (Control: P30, P50 and P70; orange by-product: PB_P_30, PB_P_50 and PB_P_70.)

Figure 3 shows the variations in the height and area (%) of the control samples and the OB_P_ samples. In the control samples, a significant (*p* < 0.05) deformation in terms of height was observed (Figure 3a), as the pulp content decreased. Sample P30 showed the largest deformation, with a deviation of 12%. This can be attributed to the lower elasticity of this sample, which affects its stability and fidelity to the printed shape. On the other hand, it was observed that the OB_P_ samples showed lower deformation compared to the control samples. No significant differences (*p* > 0.05) were found between the PB_P_50 and PB_P_70 samples, but differences (*p* < 0.05) were found with the PB_P_30 sample, which exhibited the lowest deformation in terms of height. This result could be due to the higher orange by-product content in these samples, especially in PB_P_30, which gives them a higher resistance to deformation in height.

In relation to area (Figure 3b), no significant differences (*p* > 0.05) were observed in the control samples, showing a deformation of 1.25% for P30 and P50, and 1.55% for P70. However, significant differences (*p* < 0.05) were found in the PB_P_30 and PB_P_70 samples compared to their respective controls. PB_P_30 showed a deformation 1.55% higher than the control, while PB_P_70 showed a deformation 1.46% lower than the control, being the sample with the lowest deformation in terms of area. The higher variability observed in the area of the PB_P_30 sample may be due to the higher orange by-product content present in this sample. This additional orange by-product content may affect gel formation and fiber sorting during the 3D printing process. As a result, the sample may exhibit a more brittle structure compared to other samples containing less orange by-product.

### 2.4. Post-3D Printing

In order to develop a functional 3D printed snack with a final fiber content of 3%, a post-treatment after 3D printing, specifically freeze-drying, was chosen. In vacuum freeze-drying, the water transformed into ice crystals inside the food matrix is directly sublimated and lost during the process. In addition, this treatment provides higher quality in terms of sensory and nutritional properties of the food compared to other drying techniques [28]. In 3D food printing, post-processing of the printed food can lead to deformation of the structure, so it is important to assess the dimensional fidelity and shape retention after post-processing [29].

**Figure 4:**
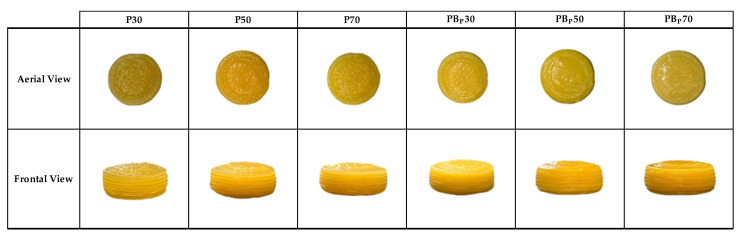
Images of the 3D printed (P) samples from aerial view and frontal view. (Control: P30, P50 and P70; orange by-product: PB_P_30, PB_P_50 and PB_P_70.)

**Figure 5:**
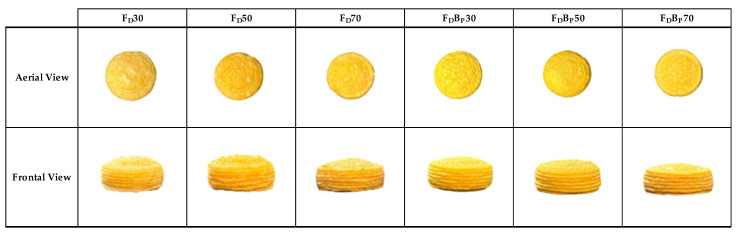
Images of the freeze-dried (F_D_) samples from aerial view and frontal view. (Control: F_D_30, F_D_ 50 and F_D_ 70; orange by-product: F_D_B_P_30, F_D_B_P_50 and F_D_B_P_70.)

The comparison between the images of the 3D printed samples and the freeze-dried samples observed from both an aerial and frontal perspective (Figure 4 and Figure 5) revealed shrinkage of the freeze-dried samples. Structural shrinkage was evident in these samples, primarily caused by the migration and loss of water during the freeze-drying process.

**Figure 6:**
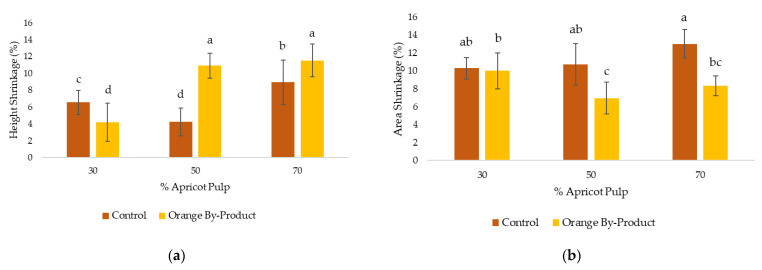
(**a**) Shrinkage of the height parameter of the freeze-dried (F_D_) samples; (**b**) shrinkage of the area parameter of the freeze-dried (F_D_) samples with respect % apricot pulp (30, 50 and 70%). Letters (a–d) indicate homogeneous groups according to ANOVA (*p* < 0.05). (Control: F_D_30, F_D_50 and F_D_70; orange by-product: F_D_B_P_30, F_D_B_P_50 and F_D_B_P_70.)

Figure 6 shows the shrinkage of the freeze-dried samples with respect to height and area (%). It was observed that samples containing a higher concentration of apricot pulp in their formulation presented a higher degree of shrinkage with respect to the height dimension (Figure 6a), being significantly higher (*p* < 0.05) in the case of sample F_D_B_P_70. This was also evident in the F_D_B_P_50 sample, with a shrinkage of the height dimension that was 6.7% higher than that of the corresponding control. In contrast, in samples with a pulp concentration of 30%, it was found that sample F_D_B_P_30 exhibited significantly less shrinkage (*p* < 0.05) compared to its control in terms of height. This phenomenon can be attributed to the presence of a higher orange by-product content within the three-dimensional network formed by the gelatin, which interferes with the total sublimation of water, inhibiting its inward retraction and thus reducing the degree of shrinkage [29,30].

In Figure 6b, a shrinkage of approximately 10% was observed for samples F_D_30 and F_D_50, and 13% for sample F_D_70, with no significant differences between them (*p* > 0.05). On the other hand, it was observed that the samples with orange by-product addition showed a lower degree of shrinkage compared to their respective controls, with the exception of the F_D_30-F_D_B_P_30 pair where no significant differences were found (*p* > 0.05). Lille et al. [31] examined samples rich in protein and cellulose, where they observed that when the initial dry matter content was high (45%), the water content was low, resulting in high structural strength.

Consequently, the freeze-dried samples with higher concentrations of pulp, and thus lower concentrations of orange by-product, exhibited a greater deformation in the height dimension (11%) compared to their controls, but not in the area (8%).

#### 2.4.1. Bite-Cutting Force

The shear force test indicates (Table 4) that exits significant differences (*p* < 0.05) between all freeze-dried gels, as a higher concentration of pulp means an increase in the maximum force to be applied to cut the samples. In addition, the orange by-product also increases the hardness of freeze-dried gels. This was reflected in a higher maximum force compared to the control samples, as was the case with the addition of pineapple co-product in low-fat beef burgers [32].

However, there are no significant differences (*p* > 0.05) between the area of F_D_30 (116 ± 21 N s) and F_D_50 (1276 ± 5 N s), indicating that the cut resistance exerted by both samples was similar.

With respect to the peaks/time ratio, it can be observed that a higher concentration of pulp increases the value of this coefficient. Likewise, the by-product contributes to increases the crispiness of the freeze-dried samples, as can be observed when comparing F_D_30 with F_D_B_P_30 and F_D_50 with F_D_B_P_50. In a study by Wójtowicz et al. [33], a corn snack was enriched with fiber and it was observed that an increase in fiber content resulted in an increase in the shear strength of the snacks. However, as the concentration of the pulp increases and that of the by-product decrease, the effect of the by-product is attenuated, as in the case of F_D_B_P_70, whose peaks/time ratio was similar to that of F_D_70. Similar results were found in freeze-dried snacks using sodium alginate and fruit pomace. Higher pomace concentration increases the hardness of the product, which means that higher shear forces are required for its destruction [34].

#### 2.4.2. Color

Table 5 indicates the values and standard deviation of the color coordinates measured in the freeze-dried gels. The results show that a higher amount of apricot pulp results in lower gel brightness, but with no significant differences (*p* > 0.05) between F_D_B_P_50 and F_D_B_P_70. The reason is that the by-product increases the brightness of the gels, but since there is a less orange by-product in the F_D_B_P_70 than in the F_D_B_P_50, the effect of the by-product is attenuated by the higher amount of pulp. As for coloration, all samples show significant differences (*p* < 0.05) among themselves, the values of a* and b* being positive in all cases. It is observed that the by-product increases the yellowish color (b*) and attenuates the reddish color (a*), with the exception of the sample F_D_B_P_30. C* indicates that the by-product increases the saturation of gels significantly (*p* < 0.05).

Regarding the color differences, ΔE1 indicates that all samples show significant differences (*p* < 0.05) with respect to the gel with the lowest concentration of apricot pulp and without by-product (F_D_30). Additionally, ΔE2 shows that the addition of by-product produces a color modification of the gels perceptible by the human eye (ΔE > 3) [35] with respect to the control samples, having significant differences (*p* < 0.05) between the three pairs of samples.

#### 2.4.3. Bioactive Compounds

Table 6 includes the mean values and standard deviations of the bioactive compounds studied in the freeze-dried gels with and without orange by-product with different percentages of apricot pulp. The use of orange by-product as a source of fiber in printed gel snacks increases the content of the bioactive compounds studied. The orange by-product showed high values for antioxidant capacity, total phenols, total carotenoids and lycopene, which were, respectively, 179 (14) mgTrolox/100 g, 858 (4) mgGA/100 g, 17.67 (0.13) mgβ-carotene/100 g and 2.62 (0.03) mg/100 g. According to the regulation no. 1924/2006 of the European Parliament and of the Council of 20 December 2006 on nutrition and health claims made in foods [15] freeze-dried gel with apricot pulp and orange by-product are a food “source of fiber” as it contains at least 3 g of fiber per 100 g.

For both groups of gels, with and without orange by-product, there is a significant (*p* < 0.05) effect of apricot pulp concentration on TC and LP content, with higher values in all cases in the samples with orange by-products. However, this behavior is only followed by the samples without orange by-products for TP, since, in the case of the samples with orange by-products, the highest TP content occurs in the samples with lower apricot pulp because they contain higher orange by-product content which presented high TP content.

AC was higher in both groups (with or without orange by-product) in the samples with higher apricot pulp content except in samples F_D_B_P_30 and F_D_B_P_50 which showed no significant difference (*p* > 0.05). AC values in F_D_70 and F_D_B_P_70 did not show significant differences (*p* > 0.05); therefore when the gel contains 70% apricot pulp, the effect of the incorporation of orange by-product is not significant (*p* > 0.05). The relationship between antioxidant capacity and bioactive compounds was higher for LP and TC, obtaining Pearson correlation coefficients of 0.8479 (*p* < 0.05) and 0.8078 (*p* < 0.05), respectively.

The sample with the lowest content of all bioactive compounds studied was F_D_30. The one with the highest TC, LP, TF and AC content was F_D_B_P_70. Considering that apricot pulp had a TP content of 81.9 (1.4) mgGA/100 g from previous studies [18], the TP content of the orange by-product is 947.1% higher than that of apricot pulp. Consequently, the samples with a higher content of orange by-product, F_D_B_P_30, exhibit higher values of TP than the rest.

## 3. Conclusions

In conclusion, the addition of orange by-product had a significant impact on both the printing process and the final quality of the snack. A predominant elastic behavior was observed in all formulations, being more pronounced in the samples with orange by-products. The pulp content and the addition of orange by-product influenced the printing process, with the samples GB_P_50 and GB_P_70 having the lowest gradient values and therefore the most difficulty to be printed. After printing, rheological changes were observed in all samples. The moduli G′ and G″ increased in the control samples but decreased in the OB_P_ samples due to a lower gel stiffness due to their higher fiber content. The addition of OB_P_ improved the printability of the gels. After the freeze-drying process, a shrinkage in height and area dimensions was observed, with a decrease of 11% and 8%, respectively, in the samples containing orange by-product. As a result, the final snack exhibited a higher crunchiness when OB_P_ was incorporated, as well as becoming a source of fiber and having a higher content of bioactive compounds (TC, LP, TP and AC). Although the samples with OB_P_ presented greater difficulty to be printed, they showed better printability both before and after lyophilization. Specifically, sample PB_P_70 showed higher structural stability and higher content of bioactive compounds. These findings highlight the potential of OB_P_ as a functional ingredient to improve both the properties and nutritional value of snacks, while contributing to the revaluation of by-products.

## 4. Materials and Methods

### 4.1. Raw Materials

Bovine gelatin was sourced from Sosa Ingredients S.L. (Barcelona, Spain), apricot pulp was provided by Jumel Alimentaria S.A. (L’Alqueria de la Comtessa, Spain), and orange by-product (B_P_) was obtained and provided by Camacho et al. [6]. The nutritional composition of the raw materials used is detailed in Table 7.

### 4.2. Formulations Gels

The control samples were made using different content of apricot pulp: 30% (G30), 50% (G50), and 70% (G70); all of them were mixed with 5% bovine gelatin (220 BLOOM). Mineral water was heated to 60 °C to dissolve the bovine gelatin. Once dissolved, it was cooled to 35 °C, and the apricot pulp was added, mixing uniformly.

The apricot pulp had a fiber content of 2.25%. Therefore, when formulating the gels with orange by-products (GB_P_30, GB_P_50 and GB_P_70), the minimum amount of orange by-product necessary to obtain a final product with 3% fiber was added, in relation to the percentage of pulp used (Table 8). The orange by-product was mixed with the gelatin tempered at 35 °C, and then the apricot pulp was added uniformly.

In both cases, the mixture was placed in 200 mL syringes and refrigerated for one hour at 4 °C. After that, the gels were tempered at room temperature (25 °C).

### 4.3. 3D Printing

This study employed a Moore 2 Pro Clay 3D printer from Shenzhen Tronxy Technology Co., Ltd., Shenzhen, China, to print control and orange by-product gels. Fused Deposition Modeling (FDM) extruder technology was utilized in the printing process, with a precise X-Y-Z positioning system and an extrusion system controlled by stepper motors. The printing was carried out at a constant temperature of 25 °C. A 3 cm diameter and 1 cm height cylinder was designed using Tinkercad (software from Autodesk, Inc., San Rafael, CA, USA), and the Ultimaker Cura software (version 5.1.1 developed by Ultimaker B.V, Brooklyn, NY, USA) was employed to set the printing parameters. The following parameters were used: a rectilinear infill of 100%, a layer height of 1.2 mm, and a speed of 20 mm/s. All samples were printed using a 1.2 mm diameter nozzle.

### 4.4. Freeze-Drying

The printed samples were frozen for 24 h at −45 °C in a vertical freezer (CVF450/45, Ing. Climas, Barcelona, Spain). Subsequently, the samples were dried in a Lioalfa-6 lyophilizer (Telstar, Spain) for 48 h at 2600 Pa and −56.6 °C.

### 4.5. Analysis

#### 4.5.1. Rheology

The Kinexus Pro + rotational rheometer (Malvern Instruments, Worcestershire, UK) was utilized for conducting oscillatory tests on gels, both before and after the printing process. The instrument was equipped with a 40 mm diameter parallel-plate geometry, featuring a 1.0 mm gap between the plates. Data acquisition and evaluation were carried out using rSpace software (Malvern Instruments, Worcestershire, UK). The amplitude sweep test was used to determine the linear viscoelastic region for all samples. The initial shear stress was varied from 0.1% to 100% at the 1 Hz frequency. An oscillatory test was performed at a fixed strain of 1 Pa and a frequency range of 0.1 to 10 Hz. All tests were performed in triplicate to ensure the reliability of the results.

#### 4.5.2. Extrusion Test

TA.XT.plus texturometer (Stable Micro Systems, Godalming, Surrey, UK) and the program Texture Exponent 32 software (Stable Micro Systems, Godalming, Surrey, UK) were used for realizing the extrusion test. To reproduce the process, the same velocity and temperature used in the printing process were applicated, maintaining the same syringe and nozzle sizes used in the preparation of the gels. The syringe with the sample and the nozzle was placed in the texturometer, stabilizing the printing plunger by means of the cylindrical press attachment. The test conditions were 10 mm distance traveled and 0.04 mm/s downstroke velocity [21]. The force/time curves allowed obtaining the parameters: gradient (Gr (N/s)), the force the plunger applies per second until reaching the maximum force; maximum force (FM (N)), the force to be applied to the sample to start extrusion through the nozzle; and mean force (Fm (N)), the force applied to continue the extrusion process.

#### 4.5.3. Image Analysis

Photographs of each printed gel and freeze-dried gel samples were taken from the front and top, and their dimensions were analyzed with ImageJ software (ImageJ, NIH, Washington, DC, USA). Front-view images were used to measure the height of the samples, while top-view images were used to measure the area of the base. To ensure accurate measurement, a metric scale of 1 cm was used as a reference. Subsequently, the percentage variation in the samples compared to the target figures was obtained as explained previously [18].

#### 4.5.4. Bite-Cutting Force

The Warner Blatzler blade (rectangular slot blade (HDP/WBR)) was incorporated into the TA.XT.plus texturometer (Stable Micro Systems, Godalming, Surrey, UK) to perform a shear force test using the Texture Exponent 32 program (Stable Micro Systems, Godalming, Surrey, UK). For the compression test, the freeze-dried samples were deposited in the center of the Heavy-Duty Platform, which has a groove that acts as a guide for the blade. The test was performed at a speed of 2 mm/s and a blade travel distance of 15 mm. The force/time curves were used to obtain the maximum force (F_M_ (N)), the area under the curve (area (N s)), and count peaks, used to obtain the peaks/time ratio (PTR (Peaks/s)).

#### 4.5.5. Color

The CIEL*a*b* color space method was used to measure the color of the freeze-dried samples. A visual angle of 10° and standard illuminant D65 was established to obtain the color coordinates using the Konica Minolta CM-700d colorimeter (Konica Minolta CM-700d/600d series, Tokyo, Japan). The parameters obtained according to the CIEL*a*b* system was L*, brightness (L* = 100 (white), L* = 0 (black)); a* (+a* = red, −a* = green,); and b* (+b* = yellow, −b* = blue) [36]. Chroma (C*ab, saturation), hue angle (h*ab). Color differences between samples were evaluated by comparing the effect of pulp in gels with and without coproduct with respect to the lowest concentration gel (F_D_30% vs. F_D_50%, F_D_70%, F_D_B_P_50%, and F_D_B_P_70%) (ΔE1), and comparing the control samples and the respective one with coproduct (F_D_30% vs. F_D_B_P_30%, F_D_50% vs. F_D_B_P_50%, and F_D_70% vs. F_D_B_P_70%) (ΔE2).

#### 4.5.6. Bioactive Compounds Determination

Total phenols, antioxidant capacity, total carotenoids, and lycopene of freeze-dried apricot gels were determined. For the analysis the freeze-dried samples were ground in a grinder (TitanMill 300 DuoClean, Grupo Cecotec Innovaciones S.L., Quart de Poblet, Valencia, Spain) The total phenols (TP) were performed by the Folin–Ciocalteu method described by Igual et al. [37], using methanol for sample extraction. Samples extracts were centrifuged for 10 min at 10,000 rpm and 4 °C in an Eppendorf centrifuge (Eppendorf, Hamburg, Germany). Afterward, 250 µL of the supernatant was mixed in a 25 mL volumetric flask with 1.25 mL of Folin–Ciocalteu reagent and stored in the dark for 8 min. After that, it was added with 3.75 mL of Na_2_CO_3_ (7.5% concentration) and stored in the dark for 2 h. With a UV-visible spectrophotometer (UV-3100PC, VWR, Leuven, Belgium), the samples’ absorbance was read at 765 nm and expressed as mg gallic acid/100 g sample (mgGA/100 g). For the antioxidant capacity (AC), 100 µL of the supernatant of centrifuge tubes mentioned above, was mixed with 3.9 mL (DPPH 2,2-diphenyl-1-picrylhydrazyl), and the absorbance was read at 515 nm, with a second measure at 5 min [37].

For the analysis of total carotenoids (TC), hexane/acetone/ethanol solvent was used to perform the extraction as indicated by the Olives-Barba et al. method [38]. Followed by quantification using the AOAC reference spectrophotometric method [39]. In a UV-3100PC spectrophotometer (VWR, Leuven, Belgium), the absorbance was read at 446 nm. β-carotene (Sigma-Aldrich, Steinheim, Germany) was used as a standard and was expressed as mg of β-carotene per 100 g of sample (mgβ-carotene/100 g). Lycopene (LP) was determined from the TC extract and the absorbance of the samples was measured at 501 nm. LP content, according to Khamis et al. [40], was expressed as mg/100 g of sample.

### 4.6. Statistical Analysis

The Statgraphics Centurion XVIII software, version 18.1.13 (Statgraphics Technologies, Inc., The Plains, VA, USA), was utilized to assess the disparities among the samples. An analysis of variance (ANOVA) was conducted with a 95% confidence level (*p* < 0.05).

## Figures and Tables

**Table 1:** Mean values (and standard deviations) of G*, complex modulus; G′, storage modulus; G″, loss modulus; Tan δ, loss tangent; and η*, apparent viscosity of gels (with and without orange by-product) before 3D printing.

	G30	G50	G70	GB_P_30	GB_P_50	GB_P_70
G* (Pa)	181 (3) ^c^	189 (6) ^c^	258 (20) ^c^	1193 (91) ^b^	1419 (54) ^a^	1510 (64) ^a^
G′ (Pa)	180 (3) ^d^	185 (6) ^d^	252 (19) ^d^	1177 (90) ^c^	1400 (54) ^b^	1494 (64) ^a^
G″ (Pa)	20.6 (0.6) ^f^	34.1 (0.3) ^e^	56 (3) ^d^	196 (12) ^c^	230 (4) ^a^	214 (4) ^b^
Tan δ	0.114 (0.006) ^e^	0.184 (0.006) ^b^	0.226 (0.006) ^a^	0.167 (0.002) ^c^	0.164 (0.005) ^c^	0.144 (0.003) ^d^
η* (Pa s)	29.1 (0.6) ^c^	30.09 (1.07) ^c^	41 (3) ^c^	190 (14) ^b^	225 (8) ^a^	240 (10) ^a^

The letters (a–f) in rows indicate the homogeneous groups according to ANOVA (*p* < 0.05). (G, gel; GB_p_: gel with by-product; 30, 50 and 70, % apricot pulp).

**Table 2:** Mean values (and standard deviations) of (Gr, gradient; F_M_, maximum force; F_m_, mean force) of gels (with and without orange by-product) before 3D printing.

	G30	G50	G70	GB_P_30	GB_P_50	GB_P_70
Gr (N/s)	474 (19) ^a^	477 (17) ^a^	526 (29) ^a^	468 (18) ^a^	390 (30) ^b^	375 (27) ^b^
F_M_ (N)	153 (15) ^d^	161 (19) ^cd^	228 (17) ^a^	192.6 (0.2) ^bcd^	195 (13) ^ab^	197 (5) ^bc^
F_m_ (N)	134 (18) ^b^	142 (23) ^b^	187 (13) ^a^	159 (12) ^ab^	162 (25) ^ab^	164 (10) ^ab^

The letters (a–d) in rows indicate the homogeneous groups according to ANOVA (*p* < 0.05). (G, gel; GB_p_: gel with by-product; 30, 50, and 70, % apricot pulp).

**Table 3:** Mean values (and standard deviations) of G*, complex modulus; G′, storage modulus; G″, loss modulus; Tan δ, loss tangent; and η*, apparent viscosity of gels (with and without orange by-product) of gels after 3D printing.

	P30	P50	P70	PB_P_30	PB_P_50	PB_P_70
G* (Pa)	677 (42) ^d^	1038 (51) ^c^	1250 (170) ^bc^	846 (87) ^d^	1301 (118) ^ab^	1394 (73) ^a^
G′ (Pa)	676 (42) ^d^	1035 (51) ^c^	1198 (170) ^bc^	834 (87) ^d^	1285 (118) ^ab^	1378 (73) ^a^
G″ (Pa)	42.6 (1.2) ^d^	88 (14) ^c^	132 (10) ^b^	146 (6) ^b^	200 (15) ^a^	213 (4) ^a^
Tan δ	0.063 (0.002) ^e^	0.086 (0.016) ^d^	0.111 (0.009) ^c^	0.176 (0.012) ^a^	0.156 (0.002) ^b^	0.155 (0.006) ^b^
η* (Pa s)	108 (7) ^d^	165 (8) ^c^	192 (27) ^bc^	135 (14) ^d^	207 (19) ^ab^	222 (12) ^a^

The letters (a–e) in rows indicate the homogeneous groups according to ANOVA (*p* < 0.05). (P, printed sample; PB_P_: printed sample with by-product; 30, 50 and 70, % apricot pulp).

**Table 4:** Mean values (and standard deviations) of cutting force parameters (F_M_, maximum force (N); area, area force/time (N s); PTR, peaks/time ratio (peaks/s)) of freeze-dried samples.

	F_D_30	F_D_50	F_D_70	F_D_B_P_30	F_D_B_P_50	F_D_B_P_70
F_M_ (N)	95(5) ^f^	138(14) ^e^	225(10) ^d^	268(6) ^c^	312(12) ^b^	409(20) ^a^
Area (N s)	116(21) ^e^	127(5) ^e^	240(30) ^d^	279(15) ^c^	365(9) ^b^	521(8) ^a^
PTR (peaks/s)	6.38(0.97) ^b^	6.9(0.6) ^ab^	7.6(0.7) ^ab^	7.6(0.6) ^ab^	7.79(1.03) ^ab^	7.3(0.4) ^a^

The letters (a–f) in rows indicate the homogeneous groups according to ANOVA (*p* < 0.05). (F_D_: freeze-dried sample; F_D_B_P_: freeze-dried with by-product sample; 30, 50 and 70% apricot pulp).

**Table 5:** Mean values and standard deviations of color parameters L*, a*, b*, C*, h* and color differences (ΔE1 and ΔE2) of freeze-dried samples.

	F_D_30	F_D_50	F_D_70	F_D_B_P_30	F_D_B_P_50	F_D_B_P_70
L* (D65)	60.6 (2.3) ^c^	58.1 (0.8) ^d^	55.5 (1.8) ^e^	68.91 (0.95) ^a^	64.9 (0.6) ^b^	64.4 (0.9) ^b^
a* (D65)	6.76 (0.08) ^f^	12.6 (0.3) ^b^	13.6 (0.2) ^a^	8.2 (0.4) ^e^	9.46 (0.02) ^d^	11.0 (0.3) ^c^
b* (D65)	26.4 (0.5) ^f^	40.2 (0.6) ^d^	38.4 (1.5) ^e^	48.0 (0.4) ^a^	45.4 (0.4) ^b^	43.0 (0.2) ^c^
C*	27.3 (0.5) ^f^	42.1 (0.6) ^d^	40.7 (1.5) ^e^	48.6 (0.4) ^a^	46.3 (0.4) ^b^	44.4 (0.2) ^c^
h*	1.320 (0.002) ^c^	1.266 (0.005) ^d^	1.230 (0.008) ^e^	1.402 (0.007) ^a^	1.365 (0.002) ^b^	1.321 (0.007) ^c^
ΔE 1		15.4 (0.6) ^c^	15.1 (1.8) ^c^		19.6 (0.4) ^a^	17.6 (0.6) ^b^
ΔE 2				23.2 (1.6) ^b^	9.1 (1.5) ^b^	10.6 (2.3) ^a^

The letters (a–f) in rows indicate the homogeneous groups according to ANOVA (*p* < 0.05). (F_D_: freeze-dried sample; F_D_B_P_: freeze-dried with by-product sample; 30, 50 and 70% apricot pulp).

**Table 6:** Mean values (and standard deviations) of TC, total carotenoids (mg_β-carotene_/100 g); LP, lycopene (mg/100 g); TP, total phenols (mg_GA_/100 g); AC, antioxidant capacity (mg_Trolox_/100 g) and TF, total fibre (mg/100 g) of studied samples.

Sample	TC	LP	TP	AC	TF *
F_D_30	2.50 (0.13) ^f^	0.882 (0.003) ^f^	99 (2) ^f^	51 (2) ^d^	0.738 (0.002) ^g^
F_D_50	3.1 (0.2) ^d^	1.00 (0.07) ^e^	136.3 (0.9) ^e^	75 (2) ^c^	1.270 (0.002) ^f^
F_D_70	4.8 (0.3) ^d^	1.40 (0.07) ^c^	168 (5) ^d^	99 (4) ^a^	1.823 (0.0012) ^e^
F_D_B_P_30	5.37 (0.12) ^c^	1.22 (0.02) ^d^	459 (4) ^a^	88.16 (0.12) ^b^	3.51 (0.02) ^c^
F_D_B_P_50	6.72 (0.16) ^b^	1.57 (0.04) ^b^	396 (8) ^b^	89 (3) ^b^	3.56 (0.06) ^b^
F_D_B_P_70	7.10 (0.12) ^a^	1.72 (0.04) ^a^	344 (19) ^c^	101 (3) ^a^	3.624 (0.013) ^a^

In colums, the same superscript small letter indicates homogeneous groups established using ANOVA (*p* < 0.05) (F_D_: freeze-dried sample; F_D_B_P_: freeze-dried with by-product sample; 30, 50 and 70% apricot pulp). * Estimated on the basis of the fiber content of the coproduct and apricot pulp

**Table 7:** Nutritional composition of raw materials expressed in g/100 g.

Composition	Apricot Pulp	Bovine Gelatin	Orange By-Product
Protein	0.8	85.0	4.4
Carbohydrates	8.5	0.0	51.4
Fat	0.1	0.0	0.8
Fiber	2.5	0.0	37.0
Ashes	0.1	2.0	2.8
Water	88.0	5.0	36.0

Information on apricot pulp and bovine gelatine has been provided by the supplier companies and on orange by-product by Camacho et al. [6].

**Table 8:** Composition of gels per 100 g.

Raw Materials (g)	G30	G50	G70	GB_P_30	GB_P_50	GB_P_70
Apricot pulp	30	50	70	30	50	70
Mineral water	65	45	25	59.19	40.31	21.44
Bovine gelatine	5	5	5	5	5	5
OB_P_	-	-	-	5.81	4.69	3.56

OB_p_: orange by-product; G, gel; GB_p_: gel with by-product; 30, 50, and 70, % apricot pulp.

## Data Availability

Not applicable.

## References

[1] Yoha K.S., Moses J.A. (2023). 3D Printing Approach to Valorization of Agri-Food Processing Waste Streams. Foods.

[2] Muthurajan M., Veeramani A., Rahul T., Gupta R.K., Anukiruthika T., Moses J.A., Anandharamakrishnan C. (2021). Valorization of Food Industry Waste Streams Using 3D Food Printing: A Study on Noodles Prepared from Potato Peel Waste. Food Bioprocess Technol..

[3] Wong GH C., Pant A., Zhang Y., Chua C.K., Hashimoto M., Leo C.H., Tan U.X. (2022). 3D food printing—Sustainability through food waste upcycling. Mater. Today Proc..

[4] Aesan-Agencia Española de Seguridad Alimentaria y Nutrición. https://www.aesan.gob.es/AECOSAN/web/para_el_consumidor/ampliacion/desperdicios.htm.

[5] Pant A., Ni Leam P.X., Chua C.K., Tan U.-X. (2023). Valorisation of vegetable food waste utilising three-dimensional food printing. Virtual Phys. Prototyp..

[6] Camacho M.d.M., Martínez-Lahuerta J.J., Ustero I., García-Martínez E., Martínez-Navarrete N. (2023). Composition of Powdered Freeze-Dried Orange Juice Co-Product as Related to Glucose Absorption In Vitro. Foods.

[7] Mohsin A., Hussain M.H., Zaman W.Q., Mohsin M.Z., Zhang J., Liu Z., Tian X., Rehman S.U., Khan I.M., Niazi S. (2022). Advances in sustainable approaches utilizing orange peel waste to produce highly value-added bioproducts. Crit. Rev. Biotechnol..

[8] Andrianou C., Passadis K., Malamis D., Moustakas K., Mai S., Barampouti E.M. (2023). Upcycled Animal Feed: Sustainable Solution to Orange Peels Waste. Sustainability.

[9] Godoi F.C., Prakash S., Bhandari B.R. (2016). 3D printing technologies applied for food design: Status and prospects. J. Food Eng..

[10] Ioniță-Mîndrican C.-B., Ziani K., Mititelu M., Oprea E., Neacșu S.M., Moroșan E., Dumitrescu D.-E., Roșca A.C., Drăgănescu D., Negrei C. (2022). Therapeutic Benefits and Dietary Restrictions of Fiber Intake: A State of the Art Review. Nutrients.

[11] Keerthana K., Anukiruthika T., Moses J., Anandharamakrishnan C. (2020). Development of fiber-enriched 3D printed snacks from alternative foods: A study on button mushroom. J. Food Eng..

[12] Leo C.H., Lee C.P., Foo S.Y., Tan J.C.W., Da Tan J., Ong E.S., Hashimoto M. (2022). 3D printed nutritious snacks from orange peel waste. Mater. Today Proc..

[13] Jagadiswaran B., Alagarasan V., Palanivelu P., Theagarajan R., Moses J., Anandharamakrishnan C. (2021). Valorization of food industry waste and by-products using 3D printing: A study on the development of value-added functional cookies. Futur. Foods.

[14] Feng C., Zhang M., Bhandari B., Ye Y. (2020). Use of potato processing by-product: Effects on the 3D printing characteristics of the yam and the texture of air-fried yam snacks. LWT.

[15] Regulation (EC) No. 1924/2006 of the European Parliament and of the Council of 20 December 2006 on Nutrition and Health Claims Made on Foods. *Off. J. Eur. Union*
**2006**, *404*, 9–25. https://eur-lex.europa.eu/legal-content/en/ALL/?uri=CELEX%3A32006R1924.

[16] Liu Z., Zhang M., Bhandari B., Yang C. (2018). Impact of rheological properties of mashed potatoes on 3D printing. J. Food Eng..

[17] Jiang H., Zheng L., Zou Y., Tong Z., Han S., Wang S. (2019). 3D food printing: Main components selection by considering rheological properties. Crit. Rev. Food Sci. Nutr..

[18] Molina-Montero C., Matas A., Igual M., Martínez-Monzó J., García-Segovia P. (2023). Impact of Apricot Pulp Concentration on Cylindrical Gel 3D Printing. Gels.

[19] Pulatsu E., Su J.-W., Kenderes S.M., Lin J., Vardhanabhuti B., Lin M. (2021). Effects of ingredients and pre-heating on the printing quality and dimensional stability in 3D printing of cookie dough. J. Food Eng..

[20] Martínez-Monzó J., Cárdenas J., García-Segovia P. (2019). Effect of Temperature on 3D Printing of Commercial Potato Puree. Food Biophys..

[21] Matas A., Molina-Montero M.d.C., Igual M., García-Segovia P., Martínez-Monzó J. (2022). Printability Prediction of Three Gels for 3D Food Printing. Biol. Life Sci. Forum.

[22] Bercea M. (2023). Rheology as a Tool for Fine-Tuning the Properties of Printable Bioinspired Gels. Molecules.

[23] Maldonado-Rosas R., Tejada-Ortigoza V., Cuan-Urquizo E., Mendoza-Cachú D., la Peña M.M.-D., Alvarado-Orozco J.M., Campanella O.H. (2022). Evaluation of rheology and printability of 3D printing nutritious food with complex formulations. Addit. Manuf..

[24] Kulisiewicz L., Baars A., Delgado A. (2007). Effect of high hydrostatic pressure on structure of gelatin gels. Bull. Pol. Acad. Sci. Tech. Sci..

[25] Alqahtani N.K., Ashton J., Katopo L., Jones O.A.H., Kasapis S. (2016). Effect of oat particle concentration and size distribution on the phase behaviour of mixtures with gelatin. J. Food Nutr. Res..

[26] Nijdam J.J., LeCorre-Bordes D., Delvart A., Schon B.S. (2020). A rheological test to assess the ability of food inks to form dimensionally stable 3D food structures. J. Food Eng..

[27] Zheng Z., Zhang M., Liu Z. (2021). Investigation on evaluating the printable height and dimensional stability of food extrusion-based 3D printed foods. J. Food Eng..

[28] Piskov S., Timchenko L., Grimm W.-D., Rzhepakovsky I., Avanesyan S., Sizonenko M., Kurchenko V. (2020). Effects of Various Drying Methods on Some Physico-Chemical Properties and the Antioxidant Profile and ACE Inhibition Activity of Oyster Mushrooms (*Pleurotus ostreatus*). Foods.

[29] Chen X., Zhang M., Teng X., Mujumdar A.S. (2022). Internal structure design for improved shape fidelity and crispness of 3D printed pumpkin-based snacks after freeze-drying. Food Res. Int..

[30] Oréfice R.L., Hench L.L., Clark A.E., Brennan A.B. (2001). Novel sol-gel bioactive fibers. J. Biomed. Mater. Res..

[31] Lille M., Nurmela A., Nordlund E., Metsä-Kortelainen S., Sozer N. (2018). Applicability of protein and fiber-rich food materials in extrusion-based 3D printing. J. Food Eng..

[32] Selani M.M., Shirado G.A., Margiotta G.B., Saldaña E., Spada F.P., Piedade S.M., Contreras-Castillo C.J., Canniatti-Brazaca S.G. (2016). Effects of pineapple byproduct and canola oil as fat replacers on physicochemical and sensory qualities of low-fat beef burger. Meat Sci..

[33] Wójtowicz A., Mościcki L., Oniszczuk T., Combrzyński M., Mitrus M. (2014). The Effect of Fiber Addition on the Extrusion-Cooking Stability and Quality of Enriched Corn Snacks. Teka Comm. Mot. Energetics Agric..

[34] Ciurzyńska A., Popkowicz P., Galus S., Janowicz M. (2022). Innovative Freeze-Dried Snacks with Sodium Alginate and Fruit Pomace (Only Apple or Only Chokeberry) Obtained within the Framework of Sustainable Production. Molecules.

[35] Igual M., García-Segovia P., Martínez-Monzó J. (2021). Resistant maltodextrin’s effect on the physicochemical and structure properties of spray dried orange juice powders. Eur. Food Res. Technol..

[36] Commission Internationale de l’Eclairage (CIE) (1986). Colorimetry.

[37] Igual M., García-Martínez E., Camacho M.D.M., Martínez-Navarrete N. (2015). Stability of micronutrients and phytochemicals of grapefruit jam as affected by the obtention process. Food Sci. Technol. Int..

[38] Barba A.O., Hurtado M.C., Mata M.S., Ruiz V.F., De Tejada M.L.S. (2006). Application of a UV-vis detection-HPLC method for a rapid determination of lycopene and β-carotene in vegetables. Food Chem..

[39] Horwitz W., Horwitz W. (2000). Official Methods of Analysis of AOAC International.

[40] Khamis H., Lusweti K., Mwevura H., Nyanzi S., Kiremire B.T. (2017). Quantification of Lycopene from Tomatoes and Watermelons by Using Beer-Lambert Principle. Technol. Sci. Am. Sci. Res. J. Eng..

